# Heterogeneity and Evolutionary Tunability of Escherichia coli Resistance against Extreme Acid Stress

**DOI:** 10.1128/spectrum.03757-22

**Published:** 2022-12-01

**Authors:** Stefanie Van Riet, Wubishet Tadesse, Julien Mortier, Susan Schlegel, Kenneth Simoens, Kristel Bernaerts, Alma Dal Co, Abram Aertsen

**Affiliations:** a Department of Molecular and Microbial Systems, KU Leuven, Leuven, Belgium; b Department of Environmental Microbiology, Eawag, Dübendorf, Switzerland; c Department of Environmental Systems Science, ETH Zurich, Zurich, Switzerland; d Department of Chemical Engineering, KU Leuven, Leuven, Belgium; University of Exeter

**Keywords:** *Escherichia coli*, GadE, acid resistance, evolution, population heterogeneity

## Abstract

Since acidic environments often serve as an important line of defense against bacterial pathogens, it is important to fully understand how the latter manage to mount and evolve acid resistance mechanisms. Escherichia coli, a species harboring many pathovars, is typically equipped with the acid fitness island (AFI), a genomic region encoding the GadE master regulator together with several GadE-controlled functions to counter acid stress. This study reveals that *gadE* and consequently AFI functions are heterogeneously expressed even in the absence of any prior acid stress, thereby preemptively creating acid-resistant subpopulations within a clonal E. coli population. Directed evolution efforts selecting for modulated *gadE* expression confirm that a gain-of-function mutation in the EvgS sensor kinase can constitutively upregulate *gadE* expression and concomitant acid resistance. However, we reveal that such upregulation of EvgS also causes cross-resistance to heat stress because of SafA-mediated cross-activation of the PhoPQ regulon. Surprisingly, loss of function of the *serC* gene (encoding phosphoserine/phosphohydroxythreonine aminotransferase) can also significantly upregulate *gadE* expression, acid resistance, and heat cross-resistance, although via a currently cryptic mechanism. As such, our data reveal a noisy expression of *gadE* in E. coli that is functional for the survival of sudden acid stress and that can readily be genetically tuned.

**IMPORTANCE** Acidic environments constitute one of the most important stresses for enteric bacteria and can be encountered in both natural (e.g., host gastrointestinal tract) and manmade (e.g., food processing) environments. The enteric species Escherichia coli harbors many pathovars and is well known for its ability to cope with acid stress. In this study, we uncover that E. coli’s acid fitness island (AFI), a genomic region that encodes important functions to deal with acid stress, is by default expressed in a heterogeneous manner. In fact, using microfluidics-based single-cell approaches, we further demonstrate that this heterogeneity preemptively creates a clonal subpopulation that is much better equipped to survive a sudden acid shock. In addition, we reveal that environments with recurring acid stress can readily select for mutants displaying a higher fraction of AFI-expressing cells. These new insights are important to properly understand and anticipate the survival characteristics of E. coli.

## INTRODUCTION

Acid stress curbs the growth and survival of bacterial competitors and pathogens in both natural and manmade environments (1–3). Indeed, acidification of the cytoplasm leads to protein unfolding by disrupting hydrogen bonds and salt bridges (4), decreased enzyme activity (5), reduced membrane integrity (6), and DNA damage via depurination and depyrimidation of protonated nitrogenous bases (1, 7). As a result, bacterial acid resistance mechanisms have received much attention and were in essence found to lower the intracellular proton concentration either via decarboxylation reactions or deamination reactions or through proton efflux (1, 8, 9). Decarboxylation reactions typically use imported glutamate, arginine, lysine, and ornithine and consume an intracellular proton when releasing the carboxyl group of these molecules. Deamination of amino acids such as arginine or glutamine releases ammonia, which can be used to buffer acidity (9, 10). Finally, the F_o_F_1_ ATPase proton pump can actively pump excess protons outside the cytoplasm (9, 11).

Escherichia coli, a species harboring many important pathovars (12), keeps its internal pH within a narrow range (typically between 7.4 and 7.8) even when the external pH ranges from 5.0 to 9.0 (9, 13). However, E. coli strains are also well equipped to withstand severe acid shock, since they tend to harbor a variant of the acid fitness island (AFI) (14). The AFI is a genomic region encoding important functions to counter acid stress (15), including GadA (part of the glutamate decarboxylase) and periplasmic chaperones HdeA and HdeB (that block the aggregation of unfolded periplasmic proteins). The AFI-encoded GadE protein is the major regulatory activator of this island, but it also upregulates expression of the *gadBC* operon that resides outside the AFI and whose functions concert with GadA (16, 17). Transcriptional and posttranscriptional control of *gadE* expression in turn depends on a myriad of regulators, of which the EvgSA two-component transduction system is the main activator under acidic conditions (18). More specifically, when the inner membrane-bound EvgS sensor kinase directly or indirectly senses acid stress, the EvgA response regulator becomes phosphorylated and (either directly or via induction of YdeO) activates the *gadE* promoter, after which the GadE regulon becomes upregulated (19, 20).

Although the GadE regulon has so far been documented to become upregulated in response to the encounter with acid stress (21) or at the transition to stationary phase (22), our results now reveal that noisy expression of *gadE* preemptively creates an acid-resistant subpopulation among isogenic E. coli siblings, even in the absence of any prior acid stress. Moreover, subsequent focus on evolutionary pathways able to modulate *gadE* expression indicates that AFI upregulation does not only stem from a gain-of-function mutation in the EvgSA upstream regulator, but can surprisingly also result from a loss-of-function mutation in serine metabolism. Furthermore, upregulation of *gadE* expression also seemed to coincide with cross-resistance to heat.

## RESULTS

### HdeD is a heterogeneously expressed membrane protein of E. coli MG1655.

**FIG 1:**
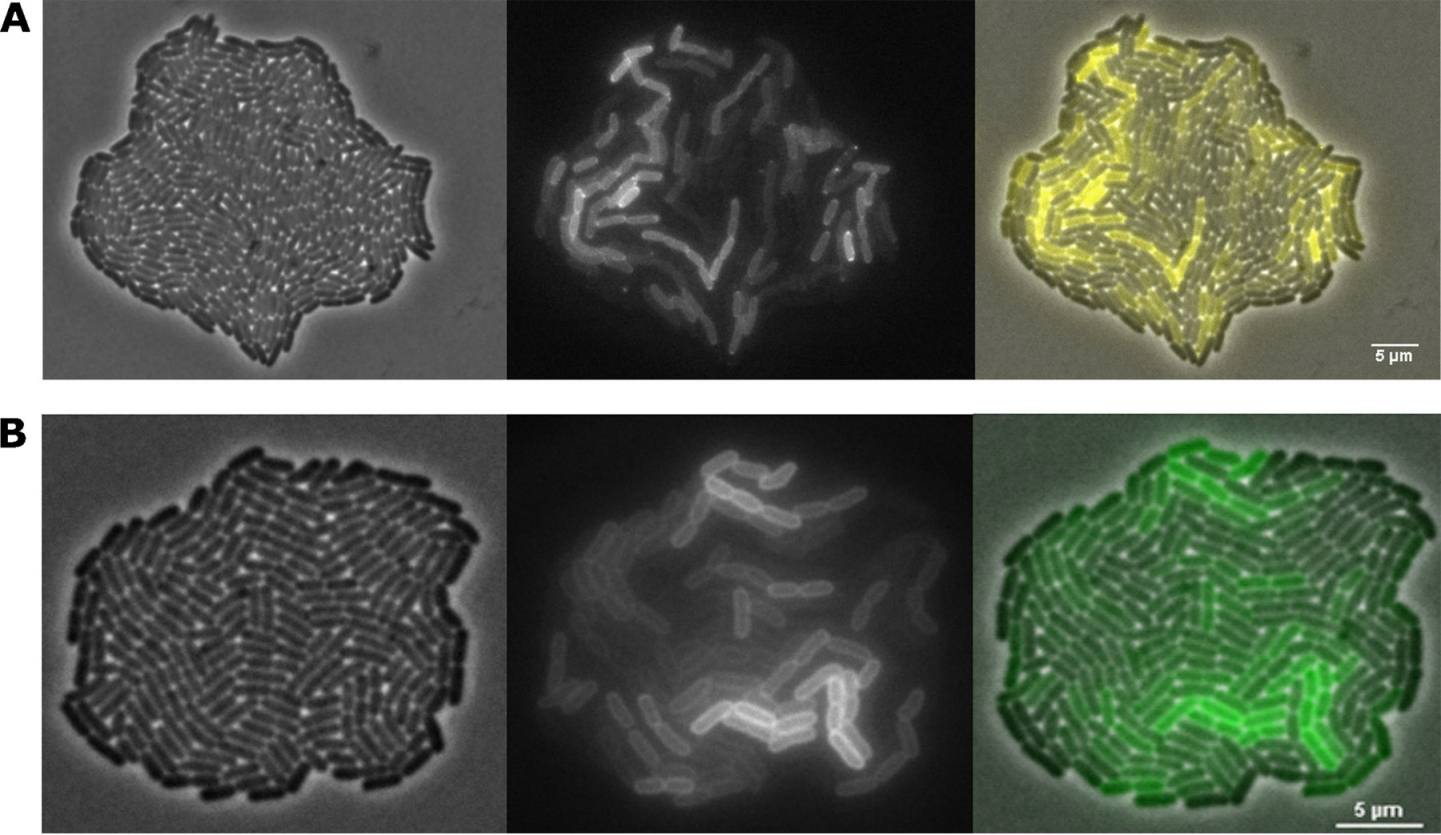
Representative phase-contrast (left panel), epifluorescence (middle panel), and superimposed (right panel) images of the original MG1655 *hdeD*::*Tn5-mVenus* transposon mutant (A) and the *de novo*-constructed MG1655 *hdeD-msfGFP* translational reporter (B) grown to microcolonies on AB agarose pads. Scale bars correspond to 5 μm.

During time-lapse fluorescence microscopy (TLFM) screening of an E. coli MG1655 fluorescent protein fusion library (using our recently described screening protocol [23] and the randomly inserting Tn*5*-*mVenus* transposon [24]), an interesting clone was found to display heterogeneous expression of what appeared to be a membrane-localized protein (Fig. 1A). Subsequent characterization of the transposon insertion site revealed that the *mVenus* reporter gene was inserted in frame in the 3′ region of the *hdeD* gene. A subsequent clean and targeted *de novo* translational fusion of the *msfGFP* gene to the 3′ end of the chromosomal *hdeD* gene (yielding MG1655 HdeD-msfGFP) confirmed that the resulting HdeD-msfGFP fusion protein displayed a similar membrane localization in the cell and heterogeneous expression throughout the population (Fig. 1B). While the membrane localization is in agreement with the study of Daley et al. (25), who showed that HdeD is an inner membrane protein, the heterogeneous expression of *hdeD* was unexpected.

### The GadE master regulator of the AFI is heterogeneously expressed.

**FIG 2:**
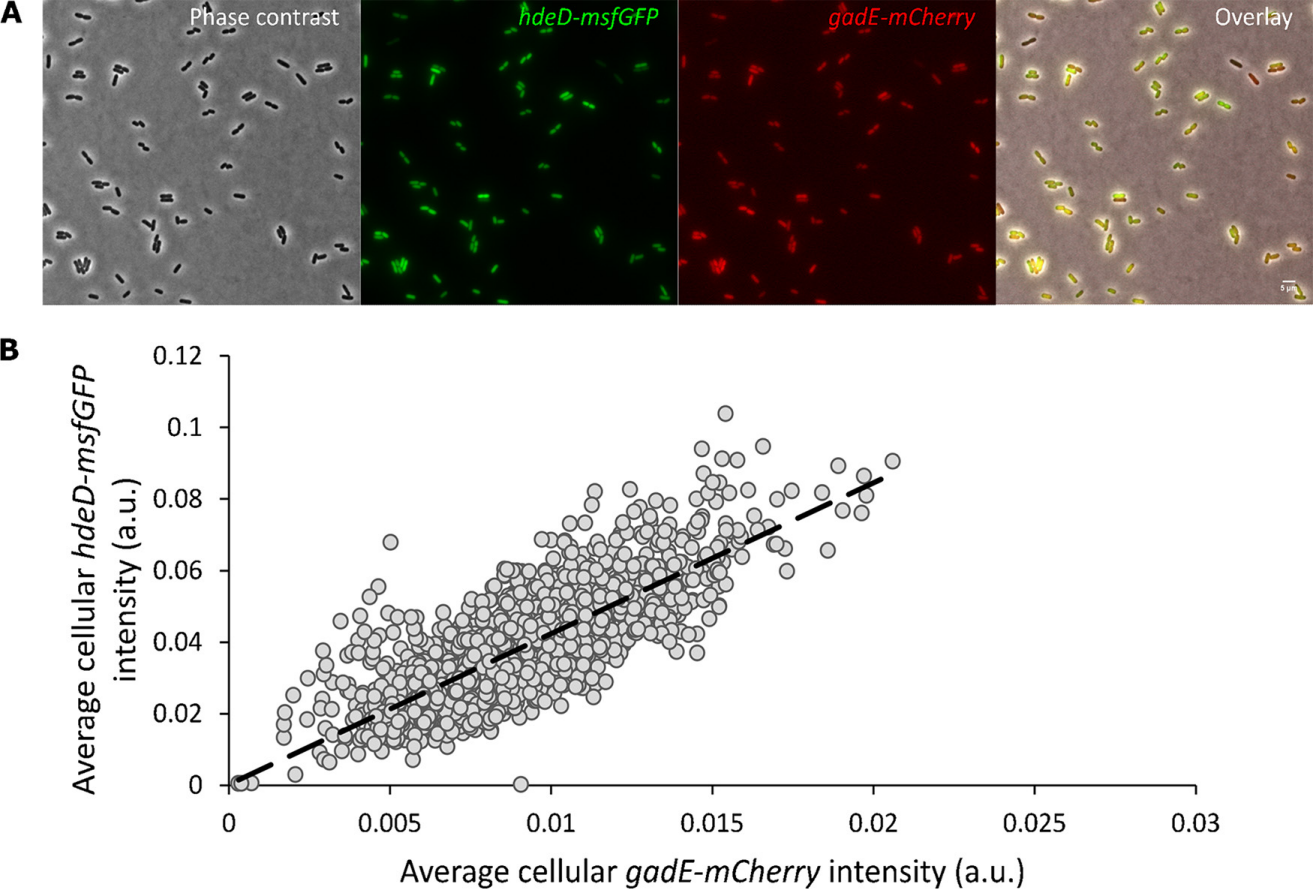
(A) Representative phase-contrast, epifluorescence, and superimposed images of the MG1655 *hdeD-msfGFP gadE-mCherry* dual transcriptional reporter grown to late exponential phase in AB medium. Scale bar corresponds to 5µm. (B) Scatter plot illustrating the statistically significant positive correlation (Spearman's rank-order correlation test, *ρ* = 0.78, *P* < 10^−16^) between *gadE-mCherry* and *hdeD-msfGFP* expression levels of individual cells (*n* = 1767) pooled from three biological replicates. The dashed line represents the best-fitting trend line, assuming a linear relationship with an intercept of zero.

The *hdeD* gene is located in the acid fitness island (AFI) of E. coli, a specific chromosomal region that consists of genes involved in pH homeostasis (15, 16), where it serves a role in repressing flagellum biosynthesis (26). Since the AFI-encoded transcriptional regulator GadE has been proposed to be the principal activator of most genes within the AFI (including the *hdeD* gene) (15, 16, 19), we wondered whether the observed *hdeD* heterogeneity was in fact indicative of a potential heterogeneity in *gadE* expression. For this, the MG1655 *hdeD-msfGFP gadE-mCherry* dual transcriptional reporter was constructed, carrying the *msfGFP* and *mCherry* reporter genes (including their own ribosome binding site) directly downstream of the *hdeD* and *gadE* open reading frames, respectively. Qualitative (Fig. 2A) and quantitative (Fig. 2B) fluorescence analyses of this dual reporter indeed revealed heterogeneous expression of *gadE*, which in turn was strongly correlated (ρ = 0.78) with the heterogeneous expression of *hdeD*. Please note that the very small cell-to-cell differences in intracellular pH within unstressed E. coli populations (ranging from 7.4 to 7.8) (13) are unlikely to drive differences in fluorophore behavior (27) or *gadE* expression (20), suggesting that the observed heterogeneity in fluorescence stems from more intrinsic regulatory effects.

### Heterogeneity in *gadE* expression generates a preexisting acid-resistant subpopulation.

**FIG 3:**
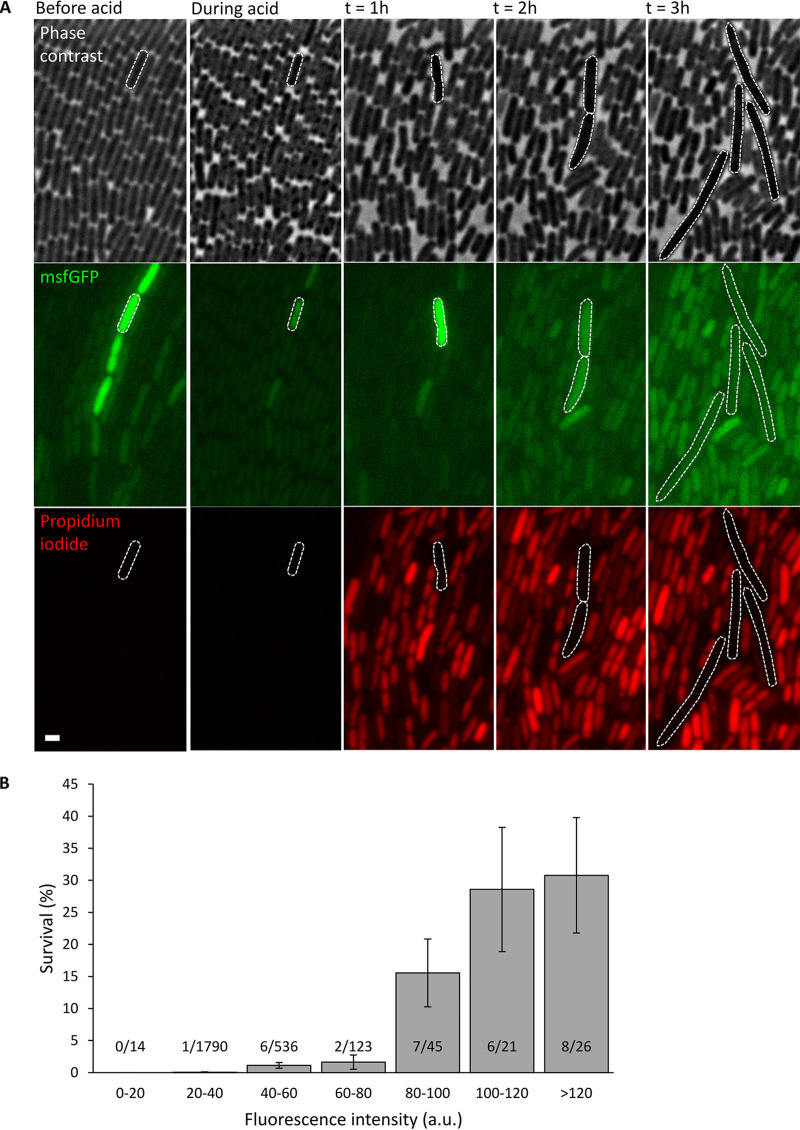
(A) Representative phase-contrast and epifluorescence images of a propidium iodide (red fluorescence)-stained clonal MG1655 *gadE-msfGFP* population growing in AB medium in a microfluidic chamber before, during, and 1 to 3 h after exposure to acid shock (pH 3 for 15 min). Scale bar corresponds to 2 µm. (B) Correlation between GFP fluorescence intensity (as a proxy of cellular *gadE* expression just before acid shock) and survival (after acid shock of pH 3 for 15 min) of cells within a clonal MG1655 *gadE-msfGFP* population. Individual cells (*n* = 2,555) were binned based on their fluorescence intensity (arbitrary units [a.u.]) displayed just before the acid shock, and bars indicate the fraction (percentage) of survivors within each bin. For each bin, its total number of cells (denominator) and its number of surviving cells (numerator) are provided as well. The graph shows representative data from two independent experiments. The effect of average cellular *msfGFP* fluorescence intensity on cell survival was found to be statistically significant (*P* < 10^−16^) by using a generalized linear mixed model where different microfluidic chambers were included as random factors. Error bars indicate bootstrapped estimates of the standard error of the fraction of surviving cells.

Since GadE is the central transcriptional activator of genes within the AFI and the *gadBC* operon (located outside the AFI and encoding the glutamate-dependent acid resistance) (15, 16, 19), we subsequently wondered whether the apparent heterogeneity of *gadE* expression could functionally cause the emergence of an acid-resistant subpopulation within a clonal E. coli population. For this, an MG1655 *gadE-msfGFP* transcriptional reporter was constructed carrying the *msfGFP* reporter gene (including its own ribosome binding site) directly downstream of the *gadE* open reading frame, after which a clonal population of this reporter strain was monitored with time-lapse fluorescence microscopy in a microfluidic device containing chambers supporting monolayer cell growth (28) (Fig. 3A and see Movie S1 in the supplemental material). After exposure to an acid shock (pH 3 for 15 min), individual cell survival was assessed by monitoring cell growth and propidium iodide uptake (staining nonsurviving cells with red fluorescence) and correlated with the *gadE* expression level (indicated via msfGFP fluorescence) of the same cells just before the acid shock. This revealed that cells stochastically displaying higher *gadE* expression levels were also much more likely to survive the acid shock (Fig. 3B), implying that the heterogeneity in *gadE* expression is functional and preemptively creates a subpopulation that is more resistant to acid stress.

Please note that during the acid shock, msfGFP fluorescence of the cells decreases (Fig. 3A) because of protonation of the fluorophore (29). After the acid shock, msfGFP fluorescence of (dying/dead) cells seems to increase again, likely because of increased cellular autofluorescence after severe stress (30) and/or as a remnant of *gadE-msfGFP* upregulation before cells succumbed and further msfGFP maturation afterwards.

### Evolutionary tunability of *gadE* expression and acid resistance.

**FIG 4:**
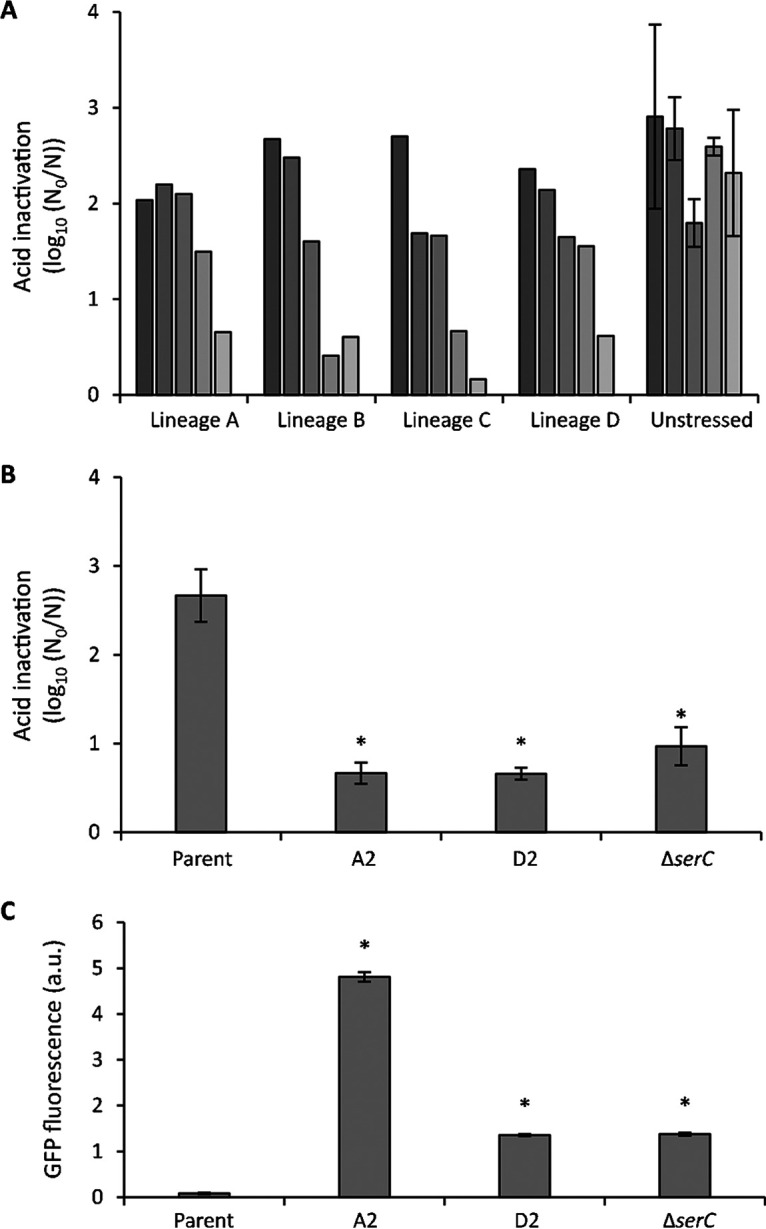
(A) Evolution toward acid resistance of MG1655 *gadE-msfGFP.* Four independent lineages (termed A to D) were iteratively exposed to acid stress (pH 2.5, 1 h) after growth to the late exponential phase in AB medium, with intermittent resuscitation and growth of survivors. As a control, three independent lineages were similarly cycled but without acid stress (unstressed). After each of the five cycles (from dark to lightly shaded bars), inactivation was monitored. For the three unstressed control lineages, the average inactivation over the three lineages is displayed together with the standard deviation. (B) Inactivation after exposure to acid (pH 2.5, 1 h) of the parental MG1655 *gadE-msfGFP* strain, its acid-evolved mutants A2 and D2, and the reconstructed MG1655 *gadE-msfGFP* Δ*serC* strain after growth to late exponential phase in AB medium. Inactivation is expressed as logarithmic reduction factor, log_10_(*N*_0_/*N*), in which *N*_0_ and *N* represent CFU per milliliter before and after acid exposure, respectively. The mean and standard deviation of results from three independent repeats are shown. (C) GFP fluorescence, measured in bulk, stemming from *gadE*-*msfGFP* expression in cultures of the parental MG1655 *gadE-msfGFP* strain, its acid-evolved mutants A2 and D2, and the reconstructed MG1655 *gadE-msfGFP* Δ*serC* strain after growth to the late exponential phase in AB medium. Fluorescence is corrected for OD_600_ and expressed in arbitrary units (a.u.). The mean and standard deviation from three independent repeats are shown. In panels B and C, an asterisk indicates statistically significant differences (*P* < 0.05) compared to the parental MG1655 *gadE-msfGFP* strain (Student’s *t* tests).

Because of the ability to preemptively (i.e., even without prior acid exposure) create acid-resistant subpopulations, we wondered whether population heterogeneity in *gadE* expression could readily be tuned by evolution. As such, a directed evolution experiment was set up in which four independent lineages (A to D) of the MG1655 *gadE-msfGFP* transcriptional reporter were iteratively exposed to acid shock (pH 2.5 for 1 h) with intermittent resuscitation and outgrowth of the survivors. This revealed that each of these lineages readily and reproducibly acquired acid resistance compared to either the parental MG1655 *gadE-msfGFP* strain or the unstressed control lineages of this reporter that were iteratively passaged without exposure to acid stress (Fig. 4A). From each of these four independent acid-resistant lineages, three random acid-resistant clones were isolated and examined for increased *gadE-msfGFP* expression compared to the parental MG1655 *gadE-msfGFP* strain. Of these 12 clones, two independent mutants (A2 and D2) displayed acid resistance in combination with significantly increased *gadE* expression levels (Fig. 4B and C).

**FIG 5:**
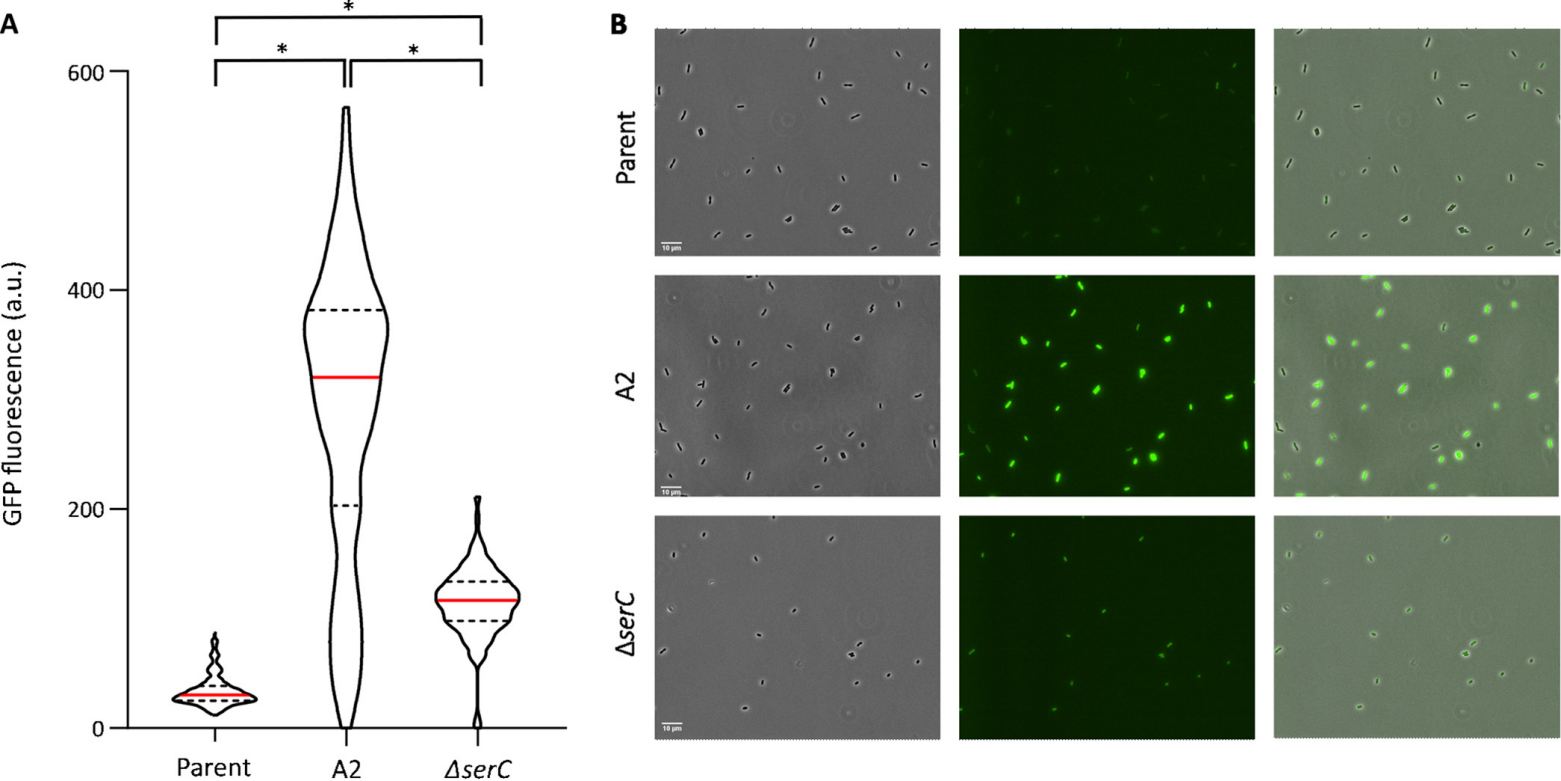
(A) Violin plots displaying the distribution of the average cellular *msfGFP* fluorescence (as a proxy of *gadE* expression and measured with fluorescence microscopy) in populations of the parental MG1655 *gadE-msfGFP* strain (*n* = 795), its acid-evolved mutant A2 (containing the *evgS*^Δ^*^564^*allele) (*n* = 636), and the reconstructed MG1655 *gadE-msfGFP* Δ*serC* strain (*n* = 561) grown to late exponential phase in AB medium. The red line within each violin plot indicates the median, while dashed lines indicate the 1st and 3rd quartiles. The distributions of *gadE* expression of the mutants are significantly different from each other and from the distribution of the parental *gadE-msfGFP* strain (Kolmogorov-Smirnov test, *P* < 0.05 [indicated by *]). (B) Representative phase-contrast (left panel), GFP epifluorescence (middle panel), and superimposed (right panel) microscopy images of the parental MG1655 *gadE-msfGFP* strain, its acid-evolved mutant A2 (containing the *evgS^Δ564^*allele), and the reconstructed MG1655 *gadE-msfGFP* Δ*serC* strain. Scale bars correspond to 10 μm.

Whole-genome sequencing of mutant A2 revealed only one mutation (Table 1), which is a 3-bp in-frame deletion within the *evgS* gene, encoding the sensor kinase of the EvgSA two-component system (31). More specifically, codon 564 (coding for arginine) within the PAS (Per-ARNT-Sim) domain (32) was lacking. Although this particular mutation has not been reported in literature so far, other subtle mutations in the PAS domain have previously been shown to bring the EvgS sensor in a constitutively activated state and to confer acid shock resistance (33–37). As such, the evolved *evgS*^Δ^*^564^* allele seems to be a gain-of-function allele as well, able to constitutively upregulate *gadE* expression. In fact, the *evgS*^Δ^*^564^* mutation imposes constitutive derepression of *gadE* expression, with the entire population being highly fluorescent (Fig. 4C and Fig. 5).

**TABLE 1:** Mutational information of acid-resistant mutants revealed by whole-genome sequencing

Mutant	Location(s)^*a*^	Gene name	Base change	Amino acid change	Gene product
A2	1689–1692	*evgS*	ΔTCG	ΔArg564	Sensor histidine kinase EvgS
D2	194	*serC*	ΔC	Ser65 followed by nonsense sequence due to frameshift	3-Phosphoserine aminotransferase
	1088	*hmp*	G→C	Gly363Ala	Nitric oxide dioxygenase

a Location refers to the position of the affected base pair starting from the start codon of the ORF.

Whole-genome sequencing of mutant D2 revealed two mutations—one in *hmp* and one in *serC* (Table 1). However, further analysis revealed that loss of function of *serC* alone was already sufficient to phenocopy the D2 mutant in terms of acid resistance and *gadE* expression (Fig. 4B and C). The *serC* gene encodes a phosphoserine/phosphodydroxythreonine aminotransferase catalyzing the reversible conversion of 3-phosphohydroxypyruvate to phosphoserine and that of 3-hydroxy-2-oxo-4-phosphonooxybutanoate to phosphohydroxythreonine in the serine and pyridoxine biosynthesis pathways, respectively (38, 39). However, so far neither SerC nor the pyridoxine and serine biosynthesis pathway has ever been linked to *gadE* expression or acid resistance. In contrast to the *evgS*^Δ^*^564^* mutation, the Δ*serC* mutation imposes a more modest upregulation of *gadE* expression (Fig. 4C and Fig. 5). Nevertheless, despite *gadE* upregulation via *evgS*^Δ^*^564^* being on average ca. 3.5-fold higher than via Δ*serC* (Fig. 4C), the levels of acid resistance of both mutants appear to be similar, which suggests that modest overexpression of the AFI is already sufficient to obtain these levels of acid resistance.

### Further characterization of *evgS*^Δ^*^564^* and Δ*serC* mutations.

**FIG 6:**
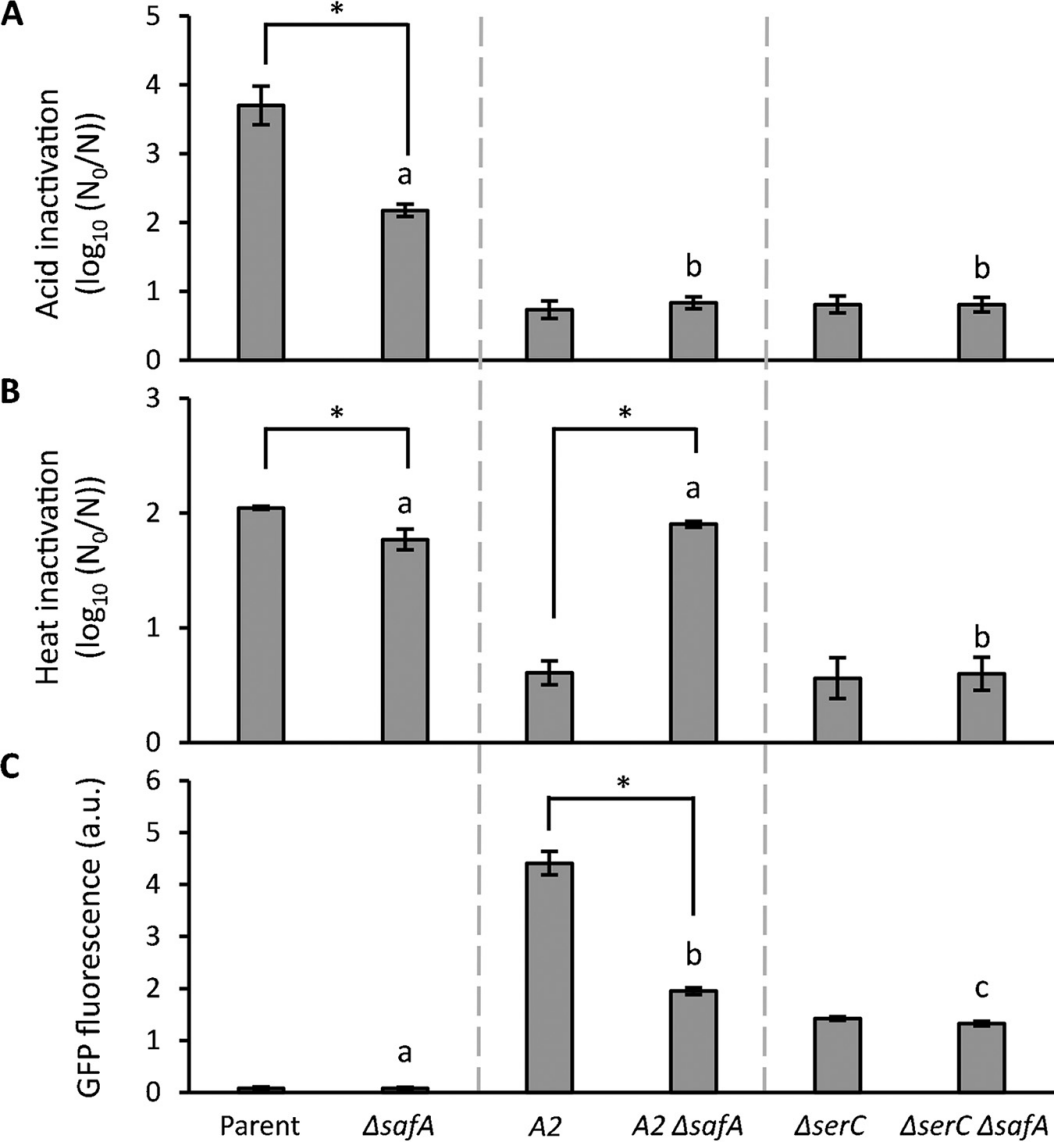
Inactivation of the parental MG1655 *gadE-msfGFP* strain, its acid-evolved mutant A2, the reconstructed MG1655 *gadE-msfGFP* Δ*serC* strain, and their corresponding Δ*safA* derivatives by acid shock (A) (pH 2.5, 1 h) or heat shock (B) (52°C, 20 min) after growth to the late exponential phase in AB medium. Inactivation is expressed as the logarithmic reduction factor, log_10_(*N*_0_/*N*), in which *N*_0_ and *N* represent CFU per milliliter before and after acid exposure, respectively. The mean and standard deviation of results from three independent repeats are shown. (C) GFP fluorescence, measured in bulk, stemming from *gadE*-*msfGFP* expression of the same strains grown to the late exponential phase in AB medium. Fluorescence is corrected for OD_600_ and expressed in arbitrary units (a.u.). The mean and standard deviation from three independent repeats are shown. An asterisk indicates statistically significant differences (*P* < 0.05) between the Δ*safA* mutant and its corresponding *safA*-proficient strain (Student’s *t* tests), and different letters indicate significant differences (*P* < 0.05) between the Δ*safA* strains (Tukey’s HSD test).

Although solely selected for acid resistance, we found that the *evgS*^Δ^*^564^* and Δ*serC* mutations also imposed a marked cross-resistance to heat (Fig. 6A and B). Constitutive genetic activation of the *evgSA* system has previously been shown to cause increased heat resistance in E. coli, although the mechanism remains elusive (34, 40). When looking at the EvgSA regulon, however, the product of the EvgA-regulated *safA* gene is known to serve as an activator of the PhoPQ two-component system, which in turn increases activity of the RpoS sigma factor that is responsible for general stress resistance (41, 42). To examine whether this coupling with the PhoPQ regulon could drive cross-resistance to heat in the MG1655 *gadE-msfGFP evgS*^Δ^*^564^* (i.e., A2) mutant, A2 was genetically deprived of its *safA* gene. This indeed revealed that (specifically) the heat resistance of the resulting MG1655 *gadE-msfGFP evgS*^Δ^*^564^* Δ*safA* mutant decreased back to the level of MG1655 *gadE-msfGFP* Δ*safA* (Fig. 6B), while its acid resistance did not become attenuated. The deletion of *safA* did partly attenuate *gadE* expression (although the resulting expression still exceeding that of its parent by 25-fold) (Fig. 6A and C), possibly because lack of SafA attenuates RpoS activity on which the *gadE* promoter depends as well. This also further confirms that modest overexpression of *gadE* is already sufficient to obtain the observed levels of acid resistance.

Interestingly, while deletion of *safA* in the Δ*serC* mutant did not significantly affect its acid resistance, it also failed to affect its heat resistance or *gadE* expression (Fig. 6). This is in sharp contrast to the impact of Δ*safA* on heat resistance and *gadE* expression of the *evgS*^Δ^*^564^* mutant and suggests that Δ*serC* is not causing its effects by upregulating the EvgSA system. However, further research should establish the impact of a Δ*evgS* mutation on the *gadE* expression and resistance of the Δ*serC* mutant.

## DISCUSSION

Although *gadE* expression and the downstream GadE regulon (including the AFI) can be triggered in response to acid stress (16), our data indicate that *gadE* expression in unstressed populations is intrinsically noisy and as such also preemptively (i.e., even in the absence of any prior acid stress) creates a subpopulation that is resistant to extreme acid stress (pH 2 to 3). Such an acid-resistant subpopulation can in particular be deterministic for the survival of low-infectious-dose pathovars of E. coli that can become faced with (sudden) extreme acid stress in the human stomach, where there might be too little time to properly sense and respond to this stress (8, 43). In addition, such heterogeneity makes the acid resistance of small populations highly variable and particularly difficult to predict based on studies with large bulk populations (44). Interestingly, it was previously observed that during growth at moderate acid stress (pH 4.6 to 4.8), E. coli is better off lacking the GadE regulon, despite its functions being essential for survival of extreme acid stress (pH 2 to 3) (45). Together with the fact that the pH varies significantly throughout the human intestinal tract (pH 1.5 to 3.5 in the stomach, pH 4 to 7 in the duodenum, pH 7 to 9 in the jejunum, pH 5 to 6 in the cecum, and pH 6.7 in the rectum) (46, 47), this suggests that noisy *gadE* expression might have become selected to optimize fitness throughout this pH-variable environment.

Heterogeneous expression of the glutamate, arginine, and lysine decarboxylase systems (48), as well as GadX (49), was most recently reported, although without linking this to differential acid survival. Interestingly, GadE and GadX can stimulate both their own and each other’s expression (17, 50), with such complex positive feedbacks possibly providing the underlying cause for the observed heterogeneity. Our serendipitous discovery of heterogeneous GadE and AFI expression in unstressed populations therefore complements these targeted studies and underscores the role of cellular individuality within the acid survival of E. coli, in support of bet-hedging and division-of-labor strategies. In this context, it was recently observed in E. coli that persister cells or cells bearing protein aggregates tend to experience a lower intracellular pH (51–53), and it will be interesting to examine whether this could serve as a cue to propagate intercellular differences in AFI expression and acid resistance as well.

Our results also underscore that adaptive evolution can rapidly adjust *gadE*-mediated population heterogeneity and hence readily improve resistance to extreme acid stress. In agreement with previous observations (34), we found that a gain-of-function mutation leading to constitutive activation of the EvgS sensor kinase can tremendously upregulate *gadE* expression throughout the population and enhance extreme acid resistance. However, our findings extend this observation by revealing that constitutive upregulation of EvgS activity inevitably also causes cross-resistance to heat via SafA-mediated cross-activation of the PhoPQ regulon. Surprisingly, a *serC* loss-of-function mutation can also shift the population toward increased *gadE* expression. Although this upregulation is more modest than that caused by the *evgS* mutation, the resulting increase in extreme acid resistance is equally strong. Loss of SerC also causes cross-resistance to heat, although in a SafA independent manner, suggesting that the modulation of *gadE* expression caused by lack of SerC is EvgSA independent. As a phosphoserine/phosphohydroxythreonine aminotransferase, SerC catalyzes the reversible conversion of glutamate to 2-oxoglutarate and is involved in the biosynthesis of the pyridoxal 5′-phosphate cofactor required by glutamate decarboxylase (39, 54). However, it is currently unclear whether or how these possible metabolic connections could underlie the modulation of *gadE* expression and/or heat cross-resistance.

The fact that acquisition of acid resistance tends to cause cross-resistance to heat in E. coli is troublesome, given the fact that this species harbors many foodborne pathovars (55) and that heat and acid are the predominant stresses used in food preservation (56). Especially in the upcoming minimal-processing regimens that aim to better retain food sensorial properties by depending on the synergistic combination of mild stresses, the use of acid as an extra hurdle to be able to lower heat exposure is popular (57). In this context, pathogens readily acquiring cross-resistance against acid and heat pose a liability.

Our results with *serC* also draw attention to the fact that adaptive mutations able to upregulate important stress response pathways are not necessarily to be found in its upstream regulators (such as *evgS*). In fact, this resonates with our recent insights regarding E. coli’s adaptive evolution toward heat resistance, where mutations in genes that are *in se* unrelated to the heat shock response can be as efficient in upregulating this response as gain-of-function mutations in the corresponding RpoH major regulator (58). As a result, mutations tuning the intrinsic stress resistance of bacteria can be hard to predict or identify. In contrast to antibiotic resistance, this makes it hard to infer a strain’s overall stress resistance based solely on its genome sequence.

In summary, our findings reveal that intrinsically noisy *gadE* expression in E. coli lies at the basis of clonal subpopulations with increased resistance to severe acid stress and that even readily acquirable loss-of-function mutations (in nonregulatory genes) can significantly upregulate *gadE* expression levels. In addition, our data also indicate that selection for improved acid resistance in E. coli can readily coselect for simultaneously improved heat resistance.

## MATERIALS AND METHODS

### Bacterial strains and growth conditions.

Throughout this study, E. coli K-12 strain MG1655 and its derivatives (see Table S1 in the supplemental material) were used. Cultures were typically grown aerobically overnight (ca. 16 h) to stationary phase in lysogeny broth (LB) (59) on an orbital shaker (200 rpm) at 37°C or at 30°C when required during strain construction. Late-exponential-phase cultures were obtained by diluting stationary-phase cultures 1/100 or 1/1,000 in fresh LB or AB medium (60) (supplemented with 10 μg/mL thiamine, 5 μg/mL uracil, and 0.5% Casamino Acids) and allowing growth for 3 to 4 h. When appropriate, the medium was supplemented with a final concentration of 100 μg/mL ampicillin (Fisher Scientific), 30 μg/mL chloramphenicol (Acros Organics), 50 μg/mL kanamycin (Applichem), or 0.2% arabinose (Acros Organics).

### Transposon mutant library construction and screening.

Using E. coli S17-1 *λpir* harboring pBAM1-Tn5-*mVenus* (20) as a conjugative donor, a random Tn5-*mVenus* transposon mutant library was constructed in E. coli MG1655 as a conjugative acceptor according to the protocol described previously by Passaris et al. (20). This transposon can create C-terminal *mVenus* translational fusions when randomly inserted into a gene in the correct reading frame and orientation (24). The resulting library was subsequently screened in high throughput with time-lapse fluorescence microscopy for clones displaying population heterogeneity according to the protocol described most recently by Mortier et al. (23) The location of the transposon insertion of interesting clones was determined via the method described by Kwon and Ricke (61).

### Construction of mutant strains.

All MG1655 mutant strains were constructed via lambda red-mediated homologous recombination (62). To construct transcriptional or translational fusions of *hdeD* or *gadE* with *msfGFP*, a PCR fragment was obtained from plasmid pDHL1029, which contains an *msfGFP-frt-nptI-frt* cassette, using the primers listed in Table S3. This fragment was subsequently recombineered into the correct locus using pKD46. The *frt*-flanked kanamycin resistance cassette (*nptI*) was excised by transiently equipping this strain with plasmid pCP20 expressing the Flp site-specific recombinase. Similarly, a transcriptional fusion of *gadE* with *mCherry* was constructed by recombineering a PCR fragment obtained from plasmid pDHL-*mCherry* using the primers listed in Table S3. In-frame deletion mutants of *safA* and *serC* were created via recombineering using pKD13 and the primers listed in Table S3.

### Acid shock and heat shock treatment.

For acid shock treatment, late-exponential-phase cultures grown in AB medium were centrifuged (4,000 × *g* for 5 min) and resuspended in the same volume of fresh AB medium acidified with HCl to pH 2.5, after which 500 μL of the resuspended culture was incubated at 37°C for 1 h while shaking in an orbital shaker (200 rpm). For heat shock treatment, 50 μL of a late-exponential-phase culture was transferred to a sterile PCR tube and incubated in a Biometra T3000 thermocycler (Biometra, Göttingen, Germany) at 52°C for 20 min. Afterwards, untreated (control) and stress-treated cells were serially diluted in LB and subsequently spotted (5 μL) on LB agar. After ca. 24 h of incubation at 37°C, CFU were counted and used to determine the CFU per milliliter of the sample. Inactivation was expressed as logarithmic reduction factor, which was calculated as log_10_(*N*_0_/*N*), in which *N*_0_ and *N* represent the CFU per milliliter before and after the treatment, respectively.

### Selection of acid-resistant mutants by directed evolution.

To obtain E. coli MG1655 *gadE-msfGFP* mutants with enhanced acid resistance, four independent cultures of this strain were iteratively grown in AB medium to the late exponential phase and intermittently treated with an acid shock (AB medium [pH 2.5] for 1 h). After each acid shock, an aliquot of the treated sample was inoculated 1/100 into fresh LB medium and regrown at 37°C for 18 h. Next the culture was diluted 1/100 in fresh AB medium and grown to the late exponential phase before exposing it to the subsequent acid shock. After 5 cycles of selection, three surviving clones for each of the evolved cultures were purified on LB and examined for their acid resistance. Simultaneously, three independent lineages of the parental MG1655 *gadE-msfGFP* strain were daily subcultured in the absence of acid stress to determine the effect of the serial passage itself.

### Whole-genome sequencing and mutation analysis.

Genomic DNA was isolated from overnight LB cultures using the GeneJET genomic DNA purification kit (Thermo Fisher Scientific). A 150-bp paired-end library was prepared using the Flex library prep kit (Illumina) and the Nextera DNA CD index kit (Illumina). Whole-genome sequencing of mutants was performed on an Illumina MiniSeq sequencer. Mutation analysis was performed using CLC Genomics Workbench. The sequencing reads were trimmed and mapped to the reference genome (MG1655) and analyzed for single nucleotide polymorphisms (SNPs), indels, and structural variants. Detected mutations were subsequently confirmed by Sanger sequencing.

### Bulk fluorescence measurement.

Cells from late-exponential-phase cultures grown in AB medium were centrifuged (4,000 × *g* for 5 min) and resuspended in the same volume of 0.85% KCl, after which 200 μL was transferred to a microplate well and placed in a Fluoroskan Ascent FL fluorimeter (Thermo Labsystems, Brussels, Belgium). The green fluorescent protein (GFP) fluorescence was measured at an excitation wavelength of 480 nm and an emission wavelength of 520 nm. Relative fluorescence units were obtained by dividing the fluorescence values by the optical density at 600 nm (OD_600_) of the same population measured in a Multiskan RC plate reader (Thermo Labsystems, Brussels, Belgium).

### Time-lapse fluorescence microscopy.

For time-lapse fluorescence microscopy (TLFM) (Fig. 1, 2, and 5), the appropriate dilution of a cell culture was transferred to an agarose pad containing AB medium and 2% agarose (Eurogentec, Seraing, Belgium). The agarose pad was constructed by placing a Gene Frame (Thermo Scientific) on a microscopy slide, adding medium supplemented with agarose, and covering it with a cover slide until solidification. TLFM was performed on a Ti-Eclipse inverted microscope (Nikon, Champigny-sur-Marne, France) equipped with a ×60 plan Apo λ oil objective, a TI-CT-E motorized condenser, a GFP filter (excitation [Ex], 472/30; Dm, 495; emission [Em], 520/35), an mCherry filter (Ex, 562/40; Dm, 593; Em, 641/75), a yellow fluorescent protein (YFP) filter (Ex, 500/24; Dichroic mirror D520; Em, 542/27), and a Nikon DS-Qi2 camera. A SpectraX LED illuminator (Lumencor, Beaverton, OR, USA) was used as the light source. Temperature was controlled with a cage incubator (Okolab, Ottaviano, Italy). Images were acquired using NIS-elements software (Nikon) and further handled with the open software ImageJ.

The microfluidic device used for Fig. 3 consists of a series of chambers connected to a long central flow channel and was constructed as described in reference 28. Cells were grown in AB medium until they fully filled the microfluidic chambers. Subsequently, the medium was switched to AB medium acidified with HCl (pH 3) for 15 min, before being switched back to AB medium at neutral pH. Throughout the experiment, the medium was supplemented with propidium iodide to assess cell viability. Image acquisition occurred every 3 min using the microscopic setup described in reference 28.

### Microscopy image analysis.

From fluorescence microscopy images, the average cellular fluorescence of an individual cell was defined as the average pixel fluorescence of a cell. For Fig. 2B and Fig. 5, cell segmentation was determined using the MicrobeTracker (63) software with manual curation. For Fig. 3B, cell segmentation was determined via the open-source software Ilastik (64), which was trained to robustly identify and segment bacterial cells and exclude debris and out-of-focus cells. Only the half of the chamber distal to the flow channel was included in the analysis of acid-shocked cells (i) because this allowed to minimize gradient effects and (ii) because chamber exiting of cells proximal to the flow channel prevented proper assessment of their viability. Cells were scored as survivors after the acid exposure had been completed when at least a single cell division event for that cell was observed after 4 h. Propidium iodide was used to qualitatively determine the cell viability status and confirm that the nongrowing cells had indeed lost viability. This revealed that virtually all nongrowing cells became transiently propidium iodide stained between 0 and 4 h after acid shock.

### Statistical analysis.

Statistical analyses (analysis of variance [ANOVA], *t* test, Tukey’s honestly significant difference [HSD] test, generalized linear mixed model, bootstrapping, and Kolmogorov-Smirnov test) were carried out using the open-source software R (65), and differences were regarded as significant when the *P* value was <0.05. The mean and standard deviations were typically calculated from at least three independent experiments. To estimate the standard errors of the proportion of survivors in Fig. 3B, the original data for each bin were bootstrapped (sampled with replacement) 10,000 times, which was used to calculate the standard deviation of the bootstrapped means.
